# Anatomically guided self-adapting deep neural network for clinically significant prostate cancer detection on bi-parametric MRI: a multi-center study

**DOI:** 10.1186/s13244-023-01439-0

**Published:** 2023-06-19

**Authors:** Ahmet Karagoz, Deniz Alis, Mustafa Ege Seker, Gokberk Zeybel, Mert Yergin, Ilkay Oksuz, Ercan Karaarslan

**Affiliations:** 1 Department of Computer Engineering, Istanbul Technical University, Istanbul, Turkey; 2 Artificial Intelligence and Information Technologies, Hevi AI Health, Istanbul, Turkey; 3 Department of Radiology, School of Medicine, Acibadem Mehmet Ali Aydinlar University, Istanbul, Turkey; 4 School of Medicine, Acibadem Mehmet Ali Aydinlar University, Istanbul, Turkey

**Keywords:** Deep learning, Magnetic resonance imaging, Prostate cancer

## Abstract

**Objective:**

To evaluate the effectiveness of a self-adapting deep network, trained on large-scale bi-parametric MRI data, in detecting clinically significant prostate cancer (csPCa) in external multi-center data from men of diverse demographics; to investigate the advantages of transfer learning.

**Methods:**

We used two samples: (i) Publicly available multi-center and multi-vendor Prostate Imaging: Cancer AI (PI-CAI) training data, consisting of 1500 bi-parametric MRI scans, along with its unseen validation and testing samples; (ii) In-house multi-center testing and transfer learning data, comprising 1036 and 200 bi-parametric MRI scans. We trained a self-adapting 3D nnU-Net model using probabilistic prostate masks on the PI-CAI data and evaluated its performance on the hidden validation and testing samples and the in-house data with and without transfer learning. We used the area under the receiver operating characteristic (AUROC) curve to evaluate patient-level performance in detecting csPCa.

**Results:**

The PI-CAI training data had 425 scans with csPCa, while the in-house testing and fine-tuning data had 288 and 50 scans with csPCa, respectively. The nnU-Net model achieved an AUROC of 0.888 and 0.889 on the hidden validation and testing data. The model performed with an AUROC of 0.886 on the in-house testing data, with a slight decrease in performance to 0.870 using transfer learning.

**Conclusions:**

The state-of-the-art deep learning method using prostate masks trained on large-scale bi-parametric MRI data provides high performance in detecting csPCa in internal and external testing data with different characteristics, demonstrating the robustness and generalizability of deep learning within and across datasets.

**Clinical relevance statement:**

A self-adapting deep network, utilizing prostate masks and trained on large-scale bi-parametric MRI data, is effective in accurately detecting clinically significant prostate cancer across diverse datasets, highlighting the potential of deep learning methods for improving prostate cancer detection in clinical practice.

**Graphical Abstract:**

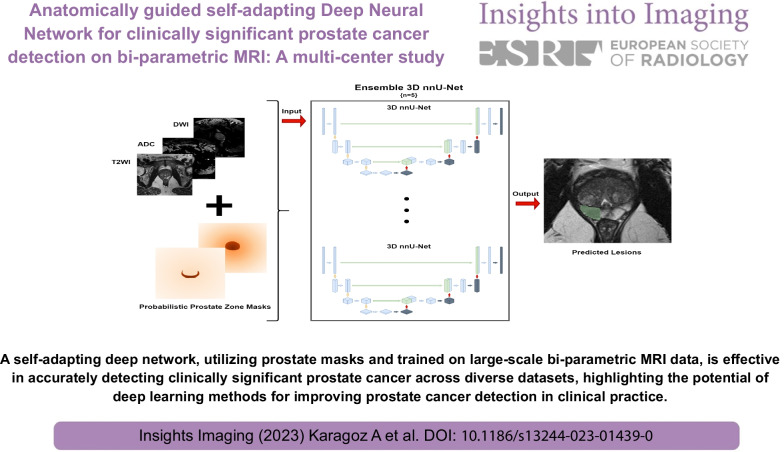

## Key points

1. We trained a self-adapting deep network on large-scale bi-parametric prostate MRI scans.
2. The model provided a high performance at detecting csPCa on in-distribution tests.
3. The performance did not drop on the external multi-center & multi-vendor data.
4. Transfer learning did not improve the performance in the external test.

## Introduction

Magnetic resonance imaging (MRI) plays an imperative role in prostate cancer (PCa) diagnostics, and the number of prostate MRI scans is expected to increase significantly as the recent evidence suggests performing pre-biopsy prostate MRI in men with suspicion of PCa [1]. The main objective of prostate MRI is to identify clinically significant PCa (csPCa) (i.e., Gleason Score ≥ 3 + 4) while sparring men with benign lesions or indolent PCa from unnecessary interventions or treatment.

The prostate imaging-reporting and data system (PI-RADS) was introduced in 2012 and most recently updated in 2019 as PI-RADS Version 2.1 to standardize prostate MRI acquisition and interpretation [2]. Though the benefits of the PI-RADS have been well recognized over the years, prostate MRI still suffers from intra-reader and inter-reader differences and non-negligible amounts of false-positive and false-negative results [3–5].

Deep learning (DL) has shown remarkable performance on a broad spectrum of medical imaging tasks in recent years, with prostate cancer diagnostics no exception. However, earlier studies have been hindered by several issues: (i) training, validating, and testing the DL models on the same data obtained [6–16]; (ii) having a small sample size [6–16]; (iii) insufficient details regarding the DL models and/or lack of open-source code sharing [6–12, 15]; (iv) lack of benchmarking DL models on publicly available datasets or challenges [6–13]; using the PI-RADS scores as the reference for performance estimation [16–18].

ProstateX challenge partially addressed the aforementioned problems, yet it did not have the adequate data size to train and test DL models effectively [19]. PI-CAI (Prostate Imaging: Cancer AI) is a new grand challenge encompassing over 10,000 prostate MRI scans [20]. The challenge allows researchers to design, train and test publicly available DL models on large-scale for identifying csPCa on bi-parametric prostate MRI.

We hypothesized that a state-of-the-art self-adapting deep network trained on large-scale bi-parametric MRI data using best practices could provide robust and generalizable performance in detecting csPCa when applied to large-scale multi-center and multi-vendor external datasets. To test our hypothesis, we first trained a self-adapting DL model, nnU-Net, using probabilistic prostate masks on the PI-CAI dataset and tested its performance on the hidden validation and testing set of the challenge. Then, we assessed the performance of the model on a private in-house multi-center dataset comprising men of different demographics. Further, we investigated the benefits of transfer learning on the performance using a small partition of the in-house dataset.

## Methods

### Study sample

We used two datasets in the present work: Publicly available PI-CAI training data and in-house data. The PI-CAI consists of over 10,000 bi-parametric prostate MRIs, yet only 1500 scans are publicly available. Researchers are also allowed to test their models on the hidden validation set, consisting of 100 scans, and the hidden testing set, consisting of 1000 scans. However, the hidden testing set was only available during a pre-defined period.

Acibadem Mehmet Ali Aydinlar University’s review board approved this retrospective study and waived the need for informed consent for the retrospective analysis of medical data. We reviewed consecutive patients who underwent a bi-parametric or multi-parametric prostate MRI scan due to suspicion of PCa (i.e., increased prostate-specific antigen or suspicious digital rectal examination) or active surveillance between January 2015 and December 2021 to create the in-house dataset.

All men in the in-house dataset had undergone whole-mount pathology or biopsy after the MRI scan or were MRI-negative (i.e., PI-RADS score of 1 or 2) with a minimum follow-up of 24 months without any clinical, laboratory, or imaging evidence of PCa. In addition, men who underwent a prostate MRI scan with an endorectal coil were excluded from the study, as were men with a history of any treatment for PCa or prostate operation.

### MRI protocols

The scans in the in-house dataset were obtained at nine institutions with 1.5 T (Avanto, Avanto-fit, and Aera, Siemens Healthcare, Erlangen/Germany; Signa HDxt Signa, General Electric Healthcare, Chicago/USA) or 3 T scanners (Prisma, Skyra, and Vida, Siemens Healthcare, Erlangen/Germany; Signa Premier, GE Healthcare, Chicago/USA).

All mpMRI or bi-parametric MRI protocols followed PI-RADS version 2 or 2.1. At a minimum, the bi-parametric prostate MRI protocol encompassed tri-planar T2-weighted and diffusion-weighted imaging. The diffusion-weighted imaging was performed with echo-planar imaging in axial planes with at least three b-values. Some patients had an acquired DWI with a b-value ≥ 1400 s/mm^2^, while others had calculated DWI with a b-value of 1400 s/mm^2^ following the PI-RADS. The ADC maps were calculated using a linear least-square fitting with all acquired b-value. We did not use dynamic contrast-enhanced images since the challenge organizers did not provide them. Further details of the MRI protocols were omitted for the sake of brevity.

### Ground-truth labels

The organizers provided the ground-truth labels for the PI-CAI dataset. For scans harboring csPCa, the organizers provided pixel-level annotations (i.e., lesion masks). For the remaining patients (i.e., those with indolent PCa or benign findings), the organizers only provided scan-level results. All csPCa lesions were annotated by trained investigators under the supervision of three expert radiologists.

The ground-truth labels of the in-house dataset were created following a similar method. First, all available pathology, radiology, and clinical reports of the patients were curated. Then, a radiologist (D.A.), one of the PI-CAI readers with three years of prostate MRI (≤ 150 prostate scans a year) and five years of DL experience, segmented the csPCa on bi-parametric prostate MRI scans. All the segmentations were carried out on a dedicated browser-based platform (https://matrix.md.ai). All segmentations were supervised by a senior radiologist (E.K.) with over 20 years of experience in prostate imaging (≥ 300 scans a year).

**Fig. 1:**
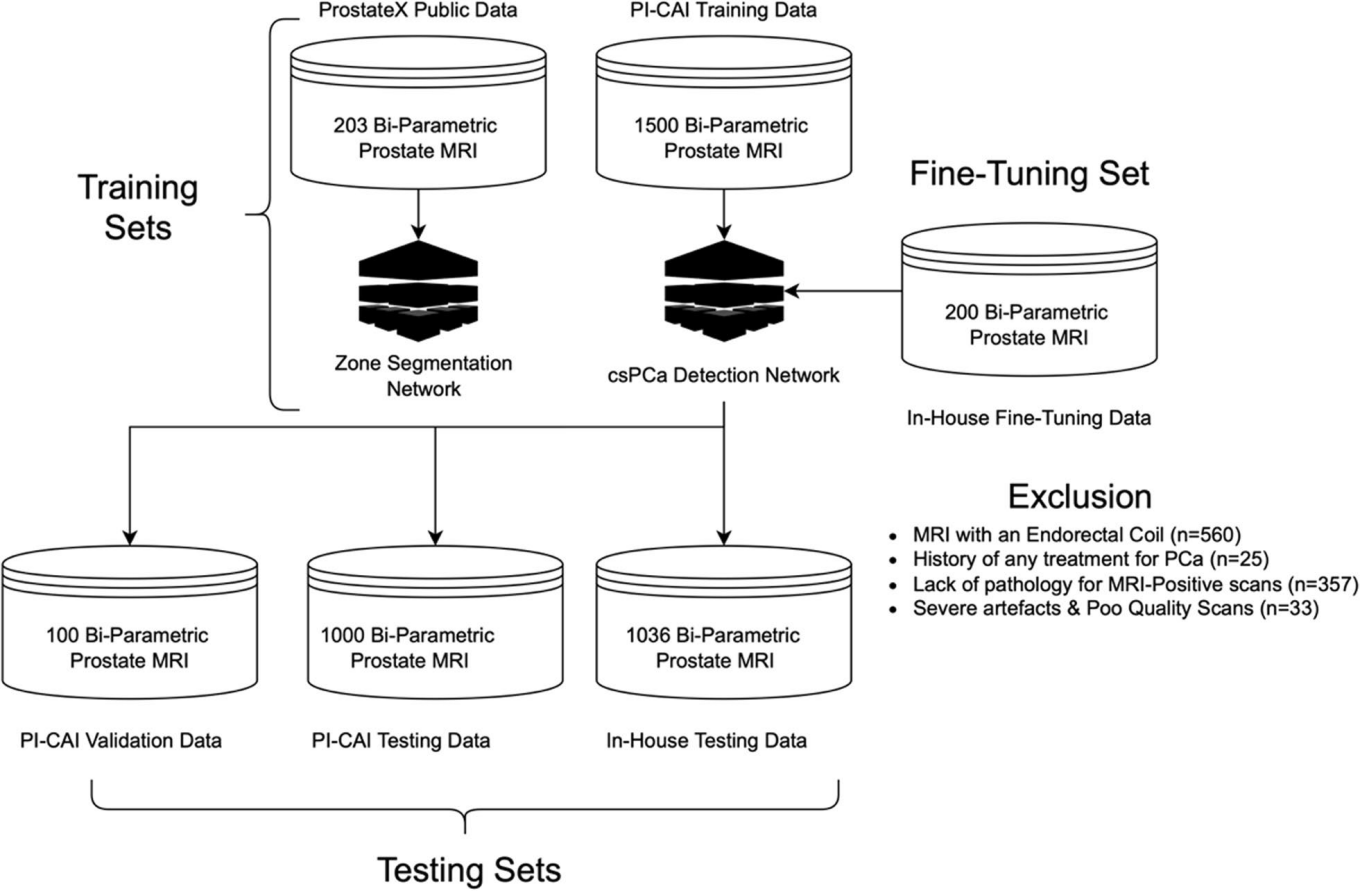
The datasets used in the study. The ProstateX data and Prostate Imaging: Cancer AI [PI-CAI] training data were used for the model training in the study. The PI-CAI data was used to train an ensemble of 3D nnU-Net models to detect clinically significant PCa

In patients with available whole-mount pathology, the radiologist determined the csPCa lesions (i.e., Gleason Score ≥ 3 + 4) using the digitized histopathological images as the reference to ensure a radio-pathological match [21]. In patients with biopsy results, the radiologist carefully read the pathology results regarding the location of the lesion. In the in-house sample, all biopsy procedures involved a combination of 3–4-core MRI/ultrasound fusion-guided biopsy followed by an extended transrectal systematic biopsy (Artemis, Eigen) [16]. Men with a benign pathology result or MRI-negative patients (i.e., those with PI-RADS 1 or 2 scans) without any clinical, laboratory, or imaging evidence of PCa were accepted as negative for csPCa. Figure 1 shows the study sample selection.

### DL models

**Fig. 2:**
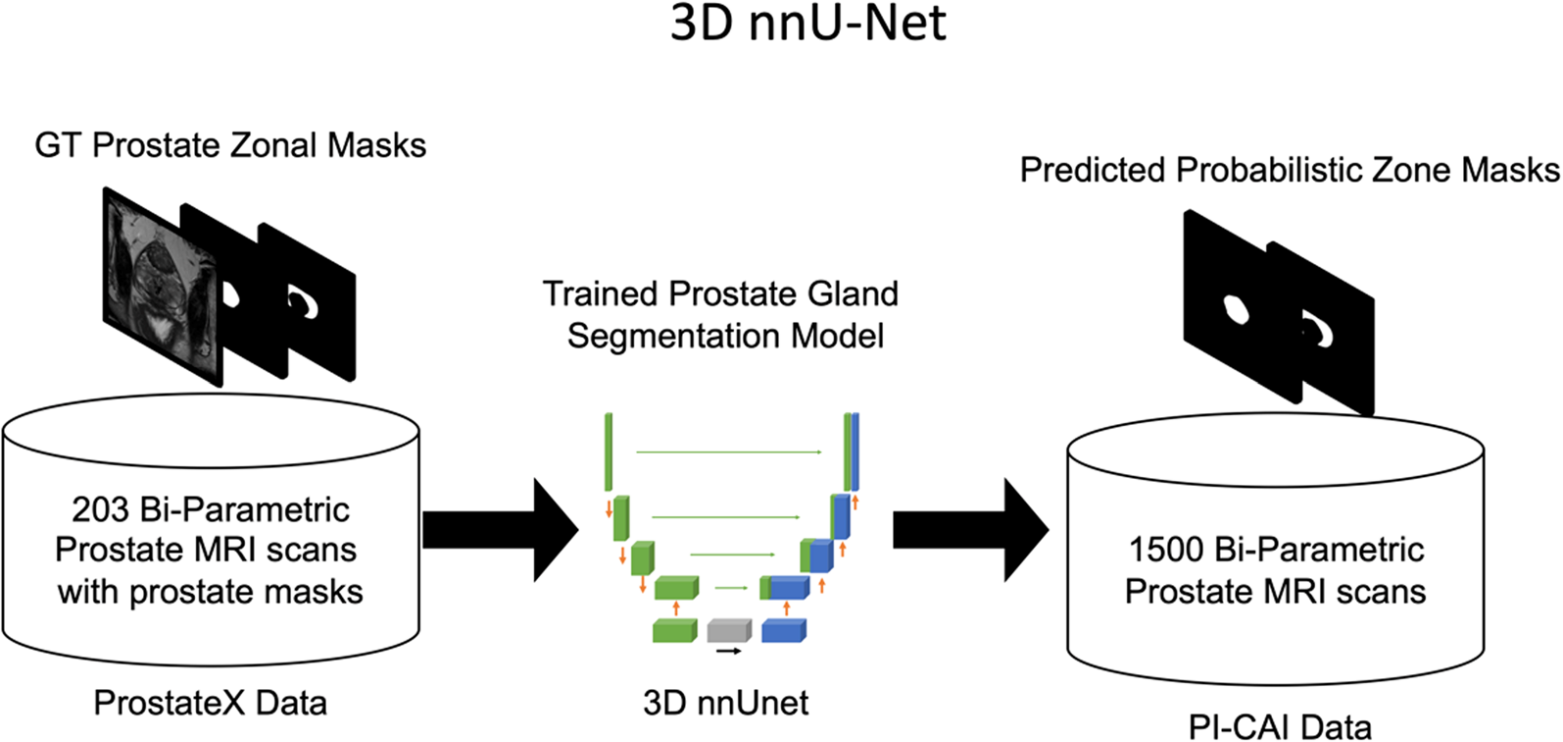
Creating the probabilistic zone masks. The ProstateX data was used to train a 3D nnU-Net for creating probabilistic prostate zone masks on the PI-CAI training data. Afterward, the probabilistic masks were used to augment the ensemble model's clinically significant prostate cancer detection performance

First, we trained a prostate zone segmentation model on patients from the publicly available ProstateX dataset [22]. We used a 3D nnU-Net, a self-adapting DL framework, fed the model with T2W images and used the peripheral and central gland masks as the ground truth [23]. Then, we used this network on challenge data to obtain probabilistic central and peripheral gland masks. Figure 2 shows an overview of the gland segmentation network.

We developed 3D nnU-Net for csPCa detection on the challenge data. We fed the model with T2W, ADC, high-b-value DWI, and probabilistic prostate segmentation masks using the csPCa masks as the ground truth.

The nnU-Net model is based on a standard U-Net architecture. This U-Net consists of two sequential encoder-decoder components interconnected via skip connections and a bottleneck layer at the bottom of the model. The encoder layers reduce the spatial resolution of the input and compute representative feature maps for the task at hand, while the decoder increases the spatial resolution, preserving the representative information for precise segmentation. Skip connections between these two layers facilitate information flow and enhance the learning process. During the training process, the U-Net parameters are continuously updated, allowing the model to implicitly learn the essential geometrical and textural features required for successful segmentation of the target masks.

**Fig. 3:**
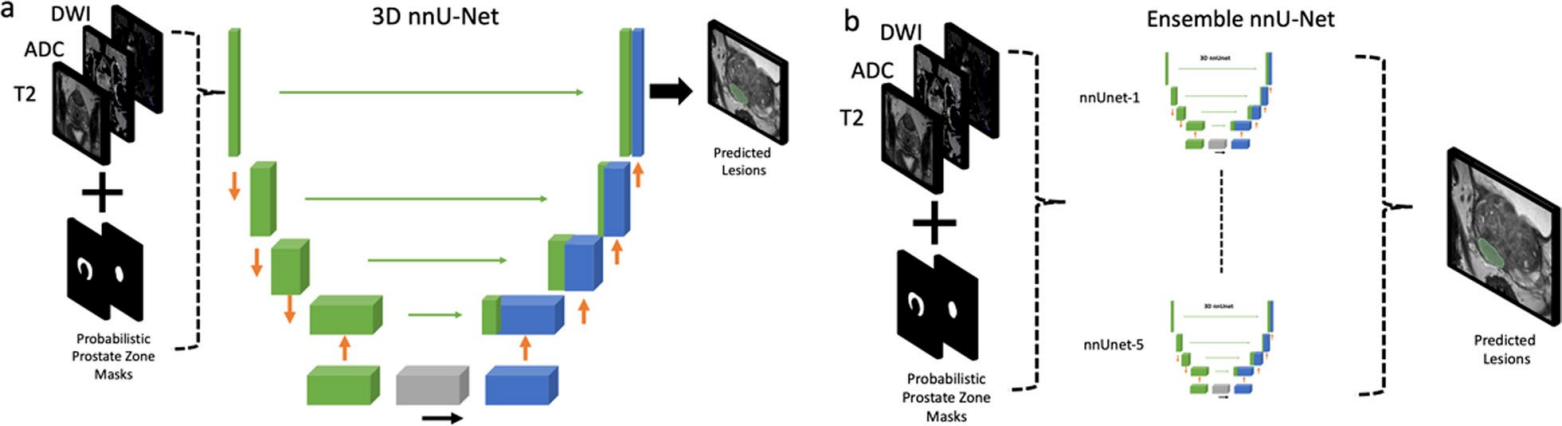
The 3D nnU-Net model for detecting clinically significant prostate cancer. **a** The 3D nnU-Net was fed with T2W imaging, diffusion-weighted imaging, and apparent diffusion coefficient maps along with probabilistic prostate masks via five different channels. The model was trained on the publicly available Prostate Imaging: Cancer AI training data using the significant cancer masks provided by the organizers as the ground truth. **b** The 3D nnU-Net model was trained using a fivefold cross-validation approach. Then, the ensemble of five nnU-Net models was used to make the final predictions

A standardized data preparation and augmentation pipeline of the nnU-Net was used in this work. Additionally, we applied extreme data augmentation and used an ensemble of networks using fivefold cross-validation. The models were trained for a thousand epochs using the loss using a combination of focal and cross-entropy loss. Figure 3 shows the csPCa segmentation models. Further details regarding the model can be found in [24].

We split the in-house data into testing and transfer learning samples by ~ 90%/10%. First, the model trained on the challenge data was used on the in-house testing set without training. Then, we fine-tuned (i.e., transfer learning) the model with a learning rate of 10^–5^ for 100 epochs and tested its performance on the testing set of the in-house data.

### Performance evaluation and statistical analyses

The statistical analyses were performed using the SciPy library of the Python programming language. The continuous variables are presented using median and interquartile ranges, and the categorical and ordinal variables are presented with frequencies and percentages. We used the area under the receiver operating characteristic (AUROC) curve to estimate patient-level performance in detecting csPCa.

The lesion-level detection performance was evaluated using the Average precision (AP) metric. True-positive lesions were the predictions that shared a minimum overlap of 0.10 in 3D space with the ground-truth annotation following the challenge and earlier studies [20, 25]. False-positive lesions were the predictions without a sufficient overlap. We also calculated the free-receiver operating curves (FROC).

We used the permutation test [26] to assess the performance difference of the DL model on the in-house testing data with and without transfer learning. The permutation test briefly shuffles performance metrics across the model with and without fine-tuning and their instances, accounting for potential differences stemming from the training method. A *p* value less than 0.05 was accepted as showing a significant result.

## Results

A total of 1202 men were enrolled in the in-house dataset with a median age of 67 years (IQR, 59–73). The in-house dataset was split into two parts: (i) testing data consisting of 1036 scans; (ii) fine-tuning data consisting of 200 scans. In all, 288 scans in the in-house testing data had csPCa, while the remaining scans had indolent cancer or benign findings. Among 288 scans with csPCa in the in-house testing data, 275 (95.48%) had available whole-mount pathology, while the diagnosis of csPCa was made by a combination of 3–4-core MRI/ultrasound fusion-guided biopsy followed by an extended transrectal systematic biopsy in remaining 13 scans (4.52%). Of the 1,500 mpMRI scans from the PI-CAI public training data, 425 had csPCa. Further details regarding the datasets are shown in Table 1.

**Table 1:** The demographics, clinical, and imaging characteristics of the PI-CAI training and in-house data set

Variables	PI-CAI training data	PI-CAI validation data	PI-CAI testing data	In-House testing data	In-house fine-tuning data
# of Patients	1476	100	1000	1002	200
# of Scans	1500	100	1000	1036	200
Age (years)	66 (61–70)	NA	NA	68 (59–73)	67 (58–70)
Prostate Specific Antigen (ng/mL)	8.5 (6–13)	NA	NA	9.2 (5–12)	8.2 (6–10)
# of different MRI scanners	5 Siemens, 2 Philips	6 Siemens, 3 Philips	6 Siemens, 3 Philips	6 Siemens, 2 GE	6 Siemens, 2 GE
# of Centers	3	3	3	9	9
PI-RADS Category of Positive MRI lesions 3 4 5	246 438 403	NA	NA	308 188 240	63 40 46
# of Scans with Benign or Indolent PCa	1075	NA	NA	NA	150
# of Scans csPCa the	425	NA	NA	288	50
# of csPCa Lesions	465	NA	NA	342	52

All continuous variables are presented with median and interquartile ranges

*csPCa* clinically significant prostate cancer, *PI-CAI* prostate imaging: cancer AI, *PI-RADS* Prostate Imaging-Reporting and Data System

*The PI-CAI training data were obtained from Radboud University Medical Center, Ziekenhuisgroep Twente, University Medical Center Groningen, Norwegian University of Science and Technology. In-house data were obtained from Acibadem Mehmet Ali Aydinlar University’s Maslak Hospital, Altunizade Hospital, Atakent Hospital, Adana Hospital, Taksim Hospital, Kozyatagi Hospital, Kocaeli Hospital, Bodrum Hospital, Eskisehir Hospital

The nnU-Net model achieved an AUROC of 0.888 and AP of 0.732 on the hidden validation data of the PI-CAI challenge, being the 1st on the leaderboard at the time of submission. The same model had an AUROC of 0.889 and an AP of 0.614 on the hidden testing data and ranked 3rd on the leaderboard. Since the validation and testing datasets were hidden, we could not draw ROC, FROC, and RP curves for the challenge data.

**Fig. 4:**
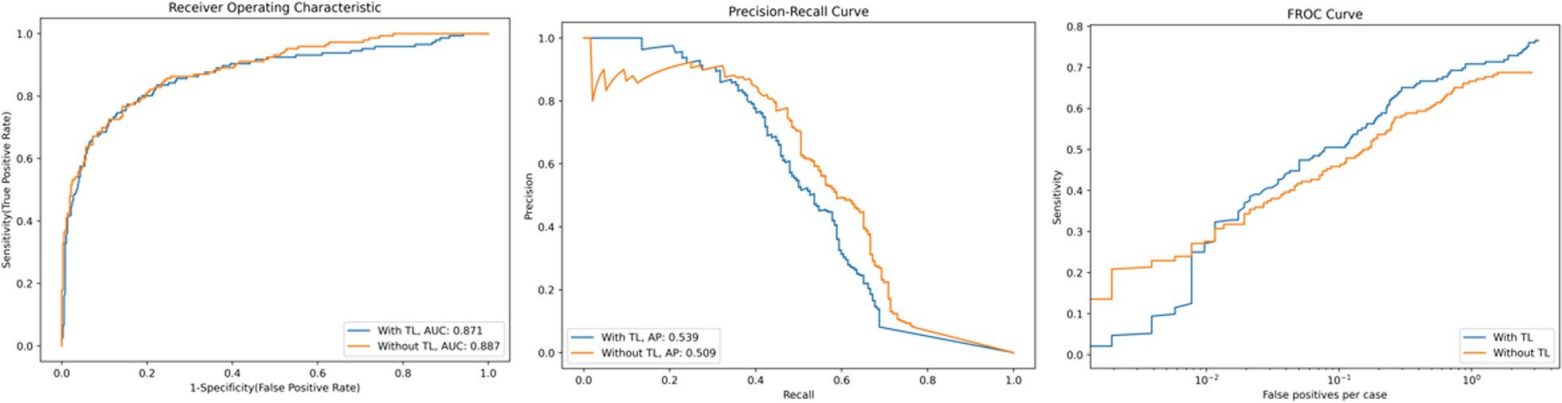
The AUROC, FROC, and PR curves of the nnU-Net in detecting clinically significant prostate cancer with and without transfer learning. The area under the receiver operating characteristic (AUROC), Free-Response Receiver Operating Characteristic (FROC), and Precision–Recall (PR) curves of the ensemble of five nnU-Net models in detecting clinically significant prostate cancer in the in-house dataset with and without transfer learning. The AUROC and FROC slightly decreased, and average precision slightly increased using transfer learning, not reaching a statistical significance

**Fig. 5:**
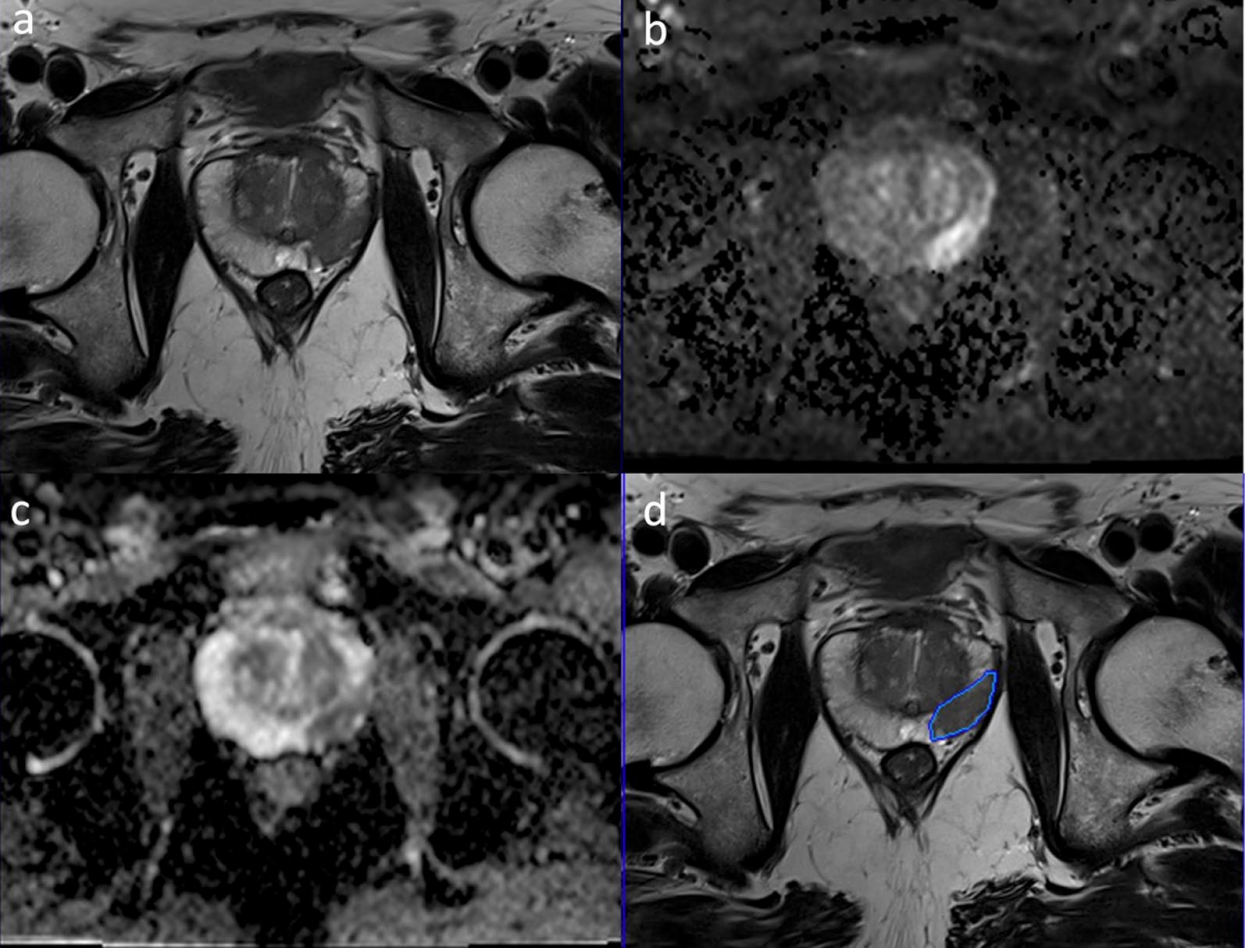
A patient with clinically significant prostate cancer in the right peripheral zone from the in-house data. The T2W (**a**), diffusion-weighted image with a b-value of 1400 s/mm^2 ^(**b**), apparent diffusion coefficient map (**c**), and the predictions of the deep learning model overlaid on the T2W image (**d**). The model correctly predicted the lesion and drew its borders

The same nnU-Net model provided an AUROC of 0.886 and AP of 0.50 on the testing part of the in-house data without fine-tuning. The AUROC of the model was similar to the challenge evaluations, yet AP showed a drop in the in-house data. After fine-tuning the model with transfer learning, the AP slightly increased to 0.539, and AUROC slightly decreased to 0.870, yet the changes were not statistically significant on average (*p* = 0.30). Figure 4 shows the AUROC, FROC, and RP curves of the nnU-Net with and without transfer learning on the in-house testing data. Figure 5 exemplifies the prediction of the model on the in-house testing data.

## Discussion

In this study, we trained a state-of-the-art self-adapting nnU-Net model using extensive data augmentations and probabilistic prostate masks on the large-scale PI-CAI data and reported its performance on the hidden validation and testing sets of the challenge. The model was externally validated on our large-scale multi-center & multi-vendor in-house data, which provided a similar performance in detecting csPCa at the scan level, showing its robustness and generalizability. Notably, transfer learning did not further increase the performance of the model, substantiating its generalizability and robustness against the data shift. Notably, the performance of our model was much higher than the reported median AUC of 0.79 in identifying csPCa in earlier studies [27].

Using testing data from the same data source with the training data, even in the presence of hold-out test sets with temporal split, is a known pitfall in DL applications to medical imaging [28]. Naturally, the performance of DL models evaluated on the same distribution can dramatically degrade when applied to external data due to many factors, including differences in the scanner, acquisition protocols, or patient demographics. Regardless of the cause, appropriate external testing is of utmost importance for performance estimation since clinical translation naturally requires similar diagnostic performance on unseen external data.

Apart from the present work, few other studies have investigated, at least partially, the performance of their DL model on unseen external test data. For example, Castillo et al. [29] trained their in-house model on a single-center data of 271 patients and tested its performance on 371 patients from three external datasets. The authors documented a significant drop in the performance during the external testing of the model. The DL model used in their work was a standard U-net segmentation model, while we implemented a state-of-the-art nnU-Net. Further, their training data was relatively small and derived from a single center. So, during the training, the model might not reach adequate robustness and generalizability. Additionally, in this work, we implemented extensive data augmentations along with probabilistic prostate masks to enhance the generalizability and robustness of the DL models.

Hosseinzadeh et al. [30] designed their DL models on data consisting of 2734 consecutive biopsy-naïve men derived from two centers. Similar to the present work, they used prostate masks to guide the neural nets. They trained their model on the data of the first center and tested its performance on the data of both centers. Hence, their test was not entirely external. Regardless, the DL model achieved an AUC of 0.85 in identifying csPCa in the external test. In contrast to the present work, the authors did not benchmark their models’ performance or publish their codes.

Mehta et al. [31] proposed to design a neural network that takes the entire prostate gland with scan-level ground-truth labels. The authors externally tested the performance of their DL model on two different datasets, yielding an AUC of 0.73 and 0.77, which were much lower than the performance of our model. Their follow-up [32] study included lesion-level annotations and zonal prostate masks. Despite lesion-level annotations, their model yielded an AUC of 0.70 on the external testing set, a significant drop from their internal model AUC of 0.85, suggesting the lack of generalizability and robustness across different data distributions. We suggest that a small training sample size, as also suggested by the authors, and the use of standard 2D U-net without extensive data augmentations might lead to low performance in identifying csPCa on the external data.

Netzer et al. [33] trained a nnU-Net on large-scale single-center in-house data. The authors split their in-house data temporally and achieved an AUC of 0.85 on a scan level. The authors also found that the performance of the model decreased from an AUC of 0.85 to 0.81 with the reduced training data size. Further, they benchmarked their models on the ProstateX challenge and achieved an AUC of 0.89. The main drawback of their study was that the study sample and ProstateX were obtained with scanners of the same manufacturer, presumably degrading the generalizability of the results. Indeed, the authors expressed their concerns about the small sample size and the abundance of potentially easier examples in the ProstateX data.

Saha et al. [34] designed an end-to-end csPCa detection network at a large scale. Following best practices, the authors derived training and testing samples from different centers. The authors implemented a 3D U-net leveraging ensemble method, focal loss, and probabilistic prostate zone masks, achieving an AUC of 0.86 on the external independent testing sample. Notably, the authors observed that the use of the ensemble method and probabilistic masks significantly boosted the generalizability of the model. The main drawback of the Saha et al. [34] was that the testing data set was obtained with the same manufacturer’s scanners as the training set.

Several limitations to the present work must be acknowledged. First, similar to the PI-CAI challenge, all patients without csPCa did not have a histopathology result in the present work. Likewise, some patients with csPCa had only a biopsy result, which is subject to errors compared with the reference whole-mount pathology. Nevertheless, all biopsies were MRI/ultrasound fusion-guided biopsies followed by an extended transrectal systematic biopsy, reducing the chances of potential sampling errors [16]. Further, we aimed to cover the scans encountered during clinical practice where a DL model needs to interpret many MRI-negative scans, which will not routinely undergo a biopsy.

Second, only MRI-visible csPCa were used for the training and model evaluation. Hence, readers must exercise caution that the DL models used in this work might miss MRI invisible lesions. This limitation might be mitigated by the registration of whole-mount pathology and MRI [35], yet it would significantly reduce the sample size and lead to selection bias by enrolling only patients with whole-mount pathology.

Third, we omitted contrast-enhanced sequences like the PI-CAI challenge. Despite mpMRI being the standard protocol for prostate imaging, evidence has recently emerged showing that the bi-parametric MRI is on par with the multi-parametric one, averting contrast usage and saving time [36, 37]. In a similar vein, we did not include clinical and laboratory findings in our DL models. Thus, creating a DL-based nomogram using prostate MRI along with clinical and laboratory for csPCa might be sought after in the future [38].

Fourth, we did not compare the performance of our DL model with that of radiologists or investigate its benefits to radiologists in reading prostate MRI. Regardless, the PI-CAI challenge organizers plan to compare the performance of the top-ranked models, including the model used in this study, with many radiologists with different experience levels worldwide. Likewise, we also plan to build a browser-based pipeline and invite radiologists with different levels of experience nationwide to read cases of our in-house dataset with and without the DL models. This will allow us to compare our model performance with radiologists in detecting csPCa and show whether it adds value to the readings of radiologists in terms of confidence, accuracy, and effectiveness.

## Conclusions

The state-of-the-art DL model trained using extensive data augmentations and probabilistic prostate masks trained on the large-scale PI-CAI data provided high performance in detecting csPCa on the hidden validation and testing sets of the challenge and large-scale multi-center and multi-vendor in-house data consisting of men with different demographics, showing its robustness and generalizability within and across datasets. Notably, implementing transfer learning using a small sample from the in-house data did not further improve the performance, supporting its generalizability and robustness against data shift.

## Data Availability

The in-house data used in this study is not available. However, the codes used in the present work can be found at https://github.com/ahmetkrgztr/HeviAI_picai. The weights of the models trained on the PI-CAI training data will be shared with the researchers in the upcoming months by the organizers of the challenge.

## Abbreviations

AP: Average precision
AUROC: The area under the receiver operating curve
csPCa: Clinically significant PCa
DL: Deep learning
FROC: Free-receiver operating curve
MRI: Magnetic resonance imaging
nnU-Net: No-new U-Net
PCa: Prostate cancer
PI-RADS: Prostate Imaging-Reporting and Data System
PR: Precision–recall curve
